# *Porphyromonas gingivalis* Lysate Induces TLR-2/4-Dependent NF-κB Activation and Inflammatory Damage in the Human Placental Barrier

**DOI:** 10.3390/ijms26199558

**Published:** 2025-09-30

**Authors:** Sebastián Araneda-Rojas, Christian Castillo, Ana Liempi, Alejandro Fernández-Moya, Jesús Guerrero-Muñoz, Sebastián Alfaro, Christian Gallardo, Rocío Arregui, Anilei Hoare, Maria Alejandra Gleisner, Marcela Hernández, Ulrike Kemmerling

**Affiliations:** 1 Instituto de Ciencias Biomédicas (ICBM), Facultad de Medicina, Universidad de Chile, Santiago 8380453, Chile; saranedar@odontologia.uchile.cl (S.A.-R.); ccastillor@uchile.cl (C.C.); anitavet@gmail.com (A.L.); alefernandez@ug.uchile.cl (A.F.-M.); jesus.guerrero@ug.uchile.cl (J.G.-M.); sebalfarolobos@gmail.com (S.A.); chris.gallardo88@gmail.com (C.G.); maria.gleisner@uchile.cl (M.A.G.); 2 Departamento de Patología y Medicina Oral y Laboratorio de Biología Periodontal, Facultad de Odontología, Universidad de Chile, Santiago 8380544, Chile; 3 Laboratory of Oral Microbiota Ecology, Faculty of Dentistry, Universidad Andres Bello, Santiago 8370133, Chile; anilei.hoare@unab.cl

**Keywords:** *Porphyromonas gingivalis*, periodontitis, placenta, NFkB signaling, cytokines

## Abstract

Periodontitis has been associated with adverse pregnancy outcomes, but the effect of oral pathogens on placental tissue and local immunity remains unclear. In this study, we investigated the response of human placental explants (HPEs) to lysates of *Porphyromonas (P.) gingivalis*, a keystone periodontal pathogen. Exposure to *P. gingivalis* induced significant histological damage and extracellular matrix degradation in placental tissue. The lysate activated the canonical NF-κB pathway, as demonstrated by increased phosphorylation of IκBα, particularly in the trophoblast. This activation was predominantly mediated by Toll-like receptor 2 (TLR-2), with partial contribution from TLR-4. Notably, TLR-2 protein levels decreased upon stimulation, while soluble (s) TLR-2 was markedly elevated in culture supernatants, suggesting receptor cleavage as a regulatory mechanism. *P. gingivalis* also triggered a robust proinflammatory cytokine secretion, including IL-1β, IL-6, IL-8, and TNF-α, with variable dependence on TLR-2 and TLR-4 signaling. These findings reveal that *P. gingivalis* components elicit a complex innate immune response in the placenta, driven by TLR-mediated NF-κB activation and modulated by sTLR-2. This study provides mechanistic insight into how periodontitis may contribute to placental inflammation and highlights potential pathways linking maternal oral health to pregnancy complications.

## 1. Introduction

Pregnancy is characterized by a unique immunological context in which the maternal immune system must tolerate the semi-allogeneic fetus while defending against pathogens. The placenta is a vital maternal-fetal interface, facilitating nutrient exchange and immune modulation [1,2]. Moreover, this organ provides signals that regulate the function of the maternal immune system and provide protective mechanisms against various pathogens, including viruses, parasites, and bacteria [3]. Therefore, the interplay between infectious agents and the placenta has significant maternal and fetal health implications, potentially leading to adverse pregnancy outcomes [4,5].

Periodontitis is a chronic inflammatory disease driven by a dysbiotic subgingival biofilm that disrupts host-microbe balance, affecting ~8.5% of adults worldwide [6].

*Porphyromonas gingivalis* (*P. gingivalis*), a Gram-negative, strict anaerobic, asaccharolytic coccobacillus, is one of the keystone periodontitis pathobionts in the dysbiotic subgingival biofilm due to its ability to subvert the host immune response and promote systemic inflammation [7]. *P. gingivalis* presents several virulence factors, including lipopolysaccharides (LPS) with atypical lipid A structures [8], and cysteine proteases, such as gingipains, which degrade epithelial junction proteins and modulate immune signaling [9,10].

These virulence factors can enter the systemic circulation, especially during increased gingival vascular permeability, such as pregnancy [11]. Notably, *P. gingivalis* DNA has been detected in human placental tissues [12], and its presence has been associated with adverse pregnancy outcomes [13,14,15]. In support of this, gingipain inhibition has been shown to mitigate *P. gingivalis*-induced preterm birth in experimental murine models [16].

The placenta is a temporary organ that separates the maternal and fetal compartments throughout pregnancy. It provides hormones, gas, nutrition, and waste exchange for the developing fetus, ensuring normal embryo-fetal growth/development [1], and is a key immunological mediator during pregnancy [4]. This organ begins to form approximately a week post-conception upon blastocyst implantation into the maternal endometrium. The trophoblast constitutes the outer blastocyst layer and develops into two main cell lineages: (i) the extravillous trophoblast involved in the embryo’s implantation into the endometrium, and (ii) the villous trophoblast, which is part of the placental barrier [1,17]. In humans, the maternal blood circulates within the intervillous space (IVS) and is in direct contact with the fetal-derived tissues. Thus, the first tissue of the placental barrier is the villous trophoblast, a specialized epithelium composed of two distinct cell types: an outer multinucleated syncytiotrophoblast (STB), which lacks intercellular junctions, and an inner mononuclear cytotrophoblast (CTB), composed of proliferative and germinative layers. A basal lamina separates the trophoblast from the underlying villous stroma (VS), the fetal connective tissue housing fetal capillaries [1,2,17].

The villous trophoblast expresses many pattern recognition receptors (PRRs), including Toll-like receptors (TLRs). TLR-2 and TLR-4 recognize lipoproteins, peptidoglycan, and LPS. Upon activation, these receptors initiate intracellular signaling cascades that converge on the MyD88 adaptor molecule, leading to phosphorylation of IκB, nuclear translocation of NF-κB, and transcription of proinflammatory cytokines such as TNF-α, IL-1β, IL-6, and IL-8 [18,19]. While essential for antimicrobial defense, this inflammatory signaling may be detrimental if dysregulated at the maternal-fetal interface, potentially compromising placental integrity and fetal health [15,20].

Human placental explants (HPEs) are tissue sections maintained in culture media in which the histological architecture, the cell-cell, and cell-ECM crosstalk are preserved. Importantly, HPEs have been used in biomedical research, including the host-pathogen interaction, for several decades [17]. However, no studies have investigated whether *P. gingivalis* can activate innate immune signaling at the human placental barrier.

Here, we show that *P. gigngivalis* lysate causes tissue damage and a decrease in TLR-2 protein expression with the release of soluble TLR-2 into the culture media, activation of the canonical NF-κB signaling, and a TLR-2/4 mediated proinflammatory and immunomodulating cytokine response. These findings suggest a mechanistic link between periodontal infection and placental inflammation and might explain adverse pregnancy outcomes in women with periodontitis.

## 2. Results

### 2.1. P. gingivalis Lysate Induces Dose-Dependent Histopathological Alterations in HPEs

**Figure 1:**
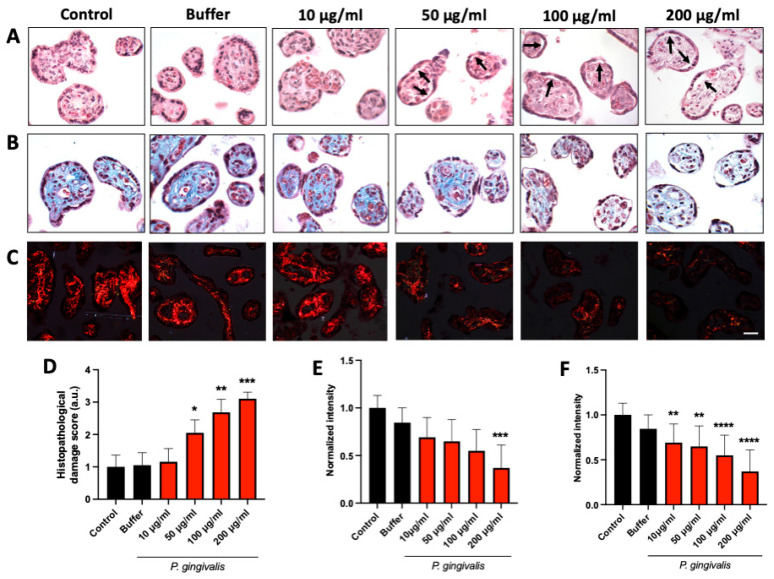
*P. gingivalis* lysate induces histopathological alterations and collagen disorganization in HPEs: HPEs were incubated for 24 h with increasing concentrations (10–200 µg/mL) of *P. gingivalis* lysate. H&E staining (**A**) revealed progressive villous damage, including trophoblast detachment (arrows) and stromal disorganization. Masson’s trichrome (**B**) and Picrosirius Red (**C**) staining showed reduced collagen deposition and disarray of collagen fibers, respectively. Semi-quantitative scoring confirmed a dose-dependent increase in histopathological damage (**D**), with significant effects at ≥50 µg/mL. Quantification of collagen content from Masson’s (**E**) and Picrosirius Red staining (**F**) demonstrated a significant loss of matrix integrity at higher lysate concentrations. Scale bar: 50 μm. Data represent mean ± SD from *n* = 3 independent placentas. * *p* < 0.05; ** *p* < 0.01; *** *p* < 0.001; **** *p* < 0.0001 versus untreated controls.

To determine the effects of *P. gingivalis* lysate on tissue integrity, HPEs were exposed to increasing concentrations (10–200 µg/mL) of lysate. Histological analysis using H&E staining revealed a concentration-dependent increase in trophoblast damage and detachment, along with marked disorganization of the villous stroma (Figure 1A,D). Quantification of histopathological damage (Table 1) showed significantly elevated scores at 50 µg/mL (* *p* < 0.05), 100 µg/mL (** *p* < 0.01), and 200 µg/mL (*** *p* < 0.001) (Figure 1D) compared to controls. Collagen integrity was also compromised in a dose-dependent manner. Masson’s trichrome staining demonstrated a progressive reduction in collagen staining intensity, with a statistically significant decrease observed at 200 µg/mL (Figure 1B,E). Additionally, analysis of collagen type I and III fibers by Picrosirius Red staining revealed a marked disorganization of collagen type I starting at 10 µg/mL, which became increasingly pronounced at higher concentrations (Figure 1C,F). These findings indicate that *P. gingivalis* lysate induces significant tissue damage, including extracellular matrix degradation, even at low doses. Based on these findings, a 50 µg/mL concentration of *P. gingivalis* lysate was selected for subsequent experiments.

### 2.2. P. gingivalis Lysate Modulates TLR-2 Expression and Induces Soluble TLR-2 Release in HPEs

**Figure 2:**
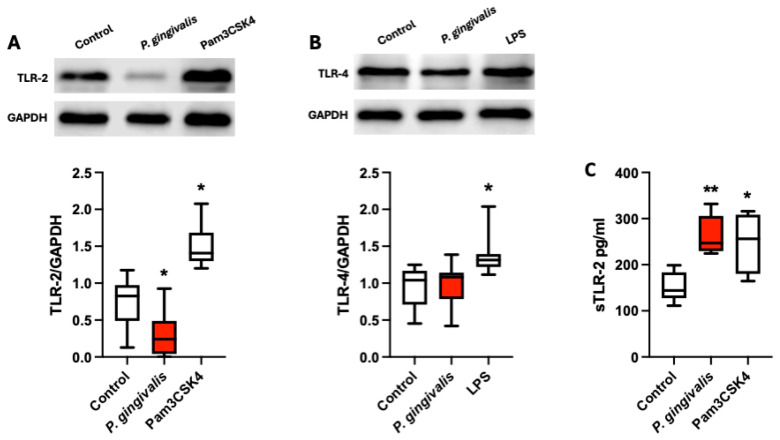
*P. gingivalis* lysate decreases TLR-2 protein levels and increases soluble TLR-2 in HPEs: HPEs were treated for 24 h with 50 µg/mL of bacterial lysate, Pam3CSK4 (1 µg/mL), or *E. coli* LPS (100 ng/mL). Western blot analysis (**A**,**B**) showed a significant reduction in TLR-2 protein levels (49.6% ± 42.7; * *p* ≤ 0.05) in lysate-treated explants, while TLR-4 expression remained unchanged. ELISA analysis of culture supernatants (**C**) revealed a marked increase in soluble TLR-2 (sTLR-2) levels in the lysate and Pam3CSK4-treated groups, suggesting receptor cleavage or release. Data represent mean ± SD from three independent experiments. * *p* < 0.05; ** *p* < 0.01 versus untreated controls.

To evaluate the impact of *P. gingivalis* lysate on TLR-2 and -4 protein expression, HPEs were incubated for 24 h, in the presence or absence, with 50 µg/mL of bacterial lysate, TLR-2 (Pam3CSK4 (1 µg/mL)), and TLR-4 (*E. coli* LPS (100 ng/mL)), agonists. Interestingly, Western blot analysis revealed a significant reduction in TLR-2 protein levels compared to unstimulated controls (*p* ≤ 0.05; Figure 2A). In contrast, TLR-4 expression remained unchanged across treatments (Figure 2B).

Given that TLR-2 can undergo post-translational proteolytic cleavage by metalloproteinases, generating sTLR-2 [21], we assessed its levels in culture supernatants. ELISA analysis showed a marked increase in sTLR-2 release following exposure to *P. gingivalis* lysate (*p* ≤ 0.05) compared to controls. A similar effect was observed in the Pam3CSK4-treated group (*p* ≤ 0.01) (Figure 2C). These findings suggest that *P. gingivalis* lysate may promote TLR-2 downregulation via cleavage and shedding, modulating the inflammatory response through the receptor availability at the maternal-fetal interface.

### 2.3. P. gingivalis Activates the Canonical NF-κB Pathway Signaling via TLR-2, but Not TLR-4, in HPEs

**Figure 3:**
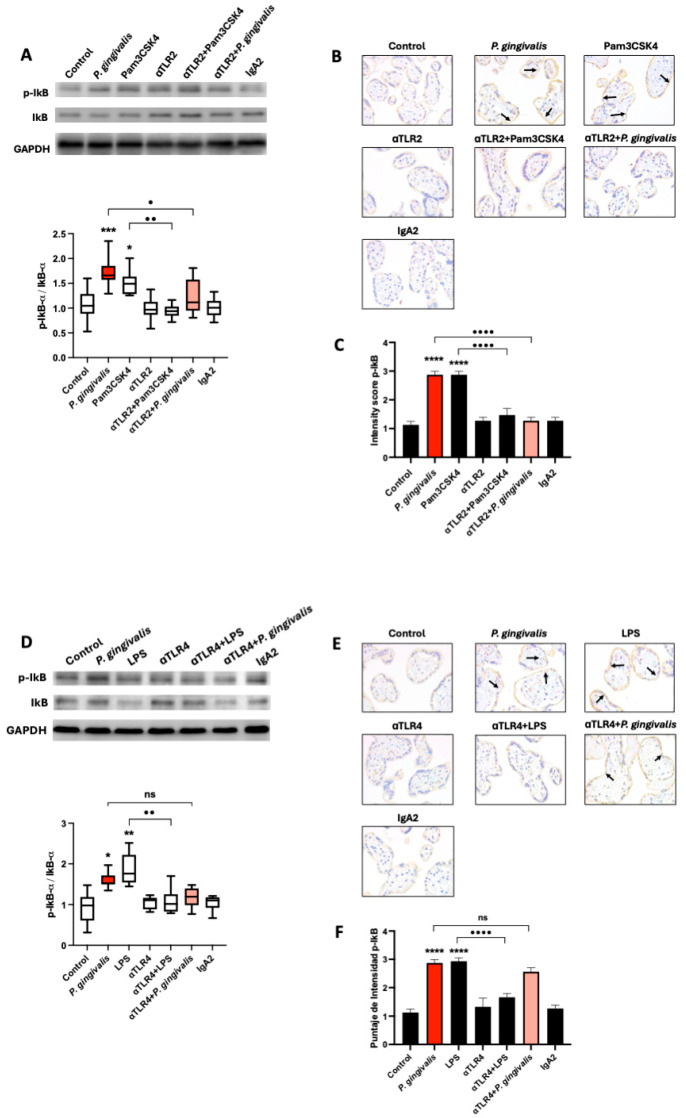
TLR-2, but not TLR-4, mediates *P. gingivalis*-induced canonical NF-κB activation in HPEs: HPEs were pre-incubated with anti-TLR-2 or anti-TLR-4 antibodies (5 µg/mL) or IgA2 isotype control for 30 min, followed by stimulation with *P. gingivalis* lysate (50 µg/mL), Pam3CSK4 (1 µg/mL), or LPS (100 ng/mL) for 10 min. Western blot analysis (**A**) demonstrated that both stimuli significantly increased the p-IκBα/total IκBα ratio, which was attenuated by TLR-2 blockade (20.0% ± 30.0 and 10.0% ± 10.0, respectively). Immunohistochemistry (**B**) localized p-IκBα expression predominantly to trophoblast cells, with quantification (**C**) confirming that TLR-2 inhibition significantly reduced p-IκBα immunoreactivity. Western blot analysis (**D**) revealed that TLR-4 neutralization significantly reduced LPS-induced NF-κB activation but did not significantly affect lysate-stimulated explants. Immunohistochemistry (**E**) and corresponding quantification (**F**) confirmed strong nuclear p-IκBα expression in trophoblasts (arrows) in both LPS and *P. gingivalis* conditions, with only the LPS response being TLR-4 dependent. Data represent mean ± SD from three independent experiments. ns: not significant; * *p* < 0.05; ** *p* < 0.01; *** *p* < 0.001; **** *p* < 0.0001 versus untreated controls.

To evaluate the involvement of TLR-2 and TLR-4 in NF-κB pathway activation (Figure 3), HPEs were incubated for 10 min with *P. gingivalis* lysate (50 µg/mL), Pam3CSK4 (1 µg/mL; TLR-2 agonist) (Figure 3), or *E. coli* LPS (100 ng/mL; TLR-4 agonist), with or without pre-incubation using neutralizing antibodies against TLR-2 (Figure 3A–C) or TLR-4 (Figure 3D–F), or an IgA2 isotype control. NF-κB activation was assessed by Western blot quantification of the p-IκBα/IκBα ratio and confirmed by immunohistochemistry for p-IκBα.

Exposure to *P. gingivalis* lysate increased the p-IκBα/IκBα ratio by 48.4% ± 21.9 (*p* ≤ 0.01), and Pam3CSK4 induced an increase of 50.8% ± 24.9. These activations were significantly inhibited by TLR-2 blockade, which reduced the p-IκBα/IκBα ratio in the *P. gingivalis* lysate condition (*p* ≤ 0.05) and in the Pam3CSK4 condition (*p* ≤ 0.01), relative to their respective ligand-only controls (Figure 3A). Neither anti-TLR-2 nor the IgA2 isotype control induced NF-κB activation alone. Immunohistochemical analysis supported these results. In the trophoblast layer, the p-IκBα staining intensity increased significantly after *P. gingivalis* exposure and after LPS treatment (both *p* ≤ 0.0001) (Figure 3B,C). Pre-treatment with anti-TLR-2 significantly reduced this response in both conditions (both *p* ≤ 0.0001) (Figure 3A–C).

In contrast, blocking TLR-4 had no significant effect on NF-κB activation induced by *P. gingivalis*, but effectively suppressed the *E. coli* LPS-induced response (Figure 3D–F). *E coli* LPS increased the p-IκBα/IκBα ratio (*p* ≤ 0.0001), which was reduced (*p* ≤ 0.0001) in the presence of anti-TLR-4 antibodies (Figure 3D) and confirmed by immunohistochemistry (Figure 3E,F).

These results confirm that *P. gingivalis* lysate activates the canonical NF-κB pathway in HPEs primarily via TLR-2, suggesting that the trophoblast is a key immune-responsive tissue at the maternal-fetal interface.

### 2.4. P. gingivalis Lysate Induces a TLR-2/4-Dependent Proinflammatory Cytokine Response in HPEs

**Figure 4:**
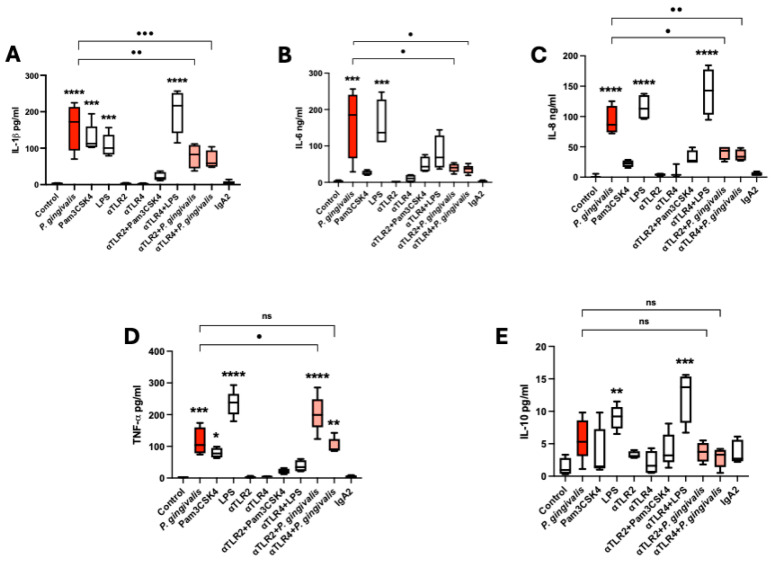
*P. gingivalis* lysate triggers a TLR-2/4-dependent cytokine response in HPEs: HPEs were incubated for 24 h with *P. gingivalis* lysate (50 µg/mL), Pam3CSK4 (1 µg/mL), or LPS (100 ng/mL), with or without pre-treatment with neutralizing antibodies against TLR-2 or TLR-4. Cytokines in supernatants were quantified by multiplex flow cytometry. *P. gingivalis* significantly increased IL-1β ((**A**); 4529.74% ± 6498.62), IL-6 ((**B**); 11.532.27% ± 9622.82), IL-8 ((**C**); 4736.04% ± 2681.8), and TNF-α ((**D**); 4183.22% ± 4374.07), with responses attenuated by TLR-2 and TLR-4 inhibition except for TNF-α, which paradoxically increased after TLR-2 blockade. *P. gingivalis* modestly elevated IL-10 levels (**E**) but was unaffected by receptor inhibition. Data represent mean ± SD from three to four independent placentas. * *p* < 0.05; ** *p* < 0.01; *** *p* < 0.001; **** *p* < 0.0001.

To evaluate the inflammatory response of placental tissue to *P. gingivalis* lysate, HPEs were incubated for 24 h with bacterial lysate (50 µg/mL), Pam3CSK4 (TLR-2 agonist), or *E. coli* LPS (TLR-4 agonist). Neutralizing antibodies against TLR-2 and TLR-4 were used to investigate receptor involvement. Cytokine levels in supernatants were quantified by multiplex flow cytometry (Figure 4).

Stimulation with *P. gingivalis* lysate significantly increased IL-1β secretion (*p* ≤ 0.0001) compared to unstimulated controls. Similar increases were observed with Pam3CSK4 (*p* ≤ 0.001) and LPS (*p* ≤ 0.001). This response by *P. gingivalis* lysate was reduced through TLR-2 (*p* ≤ 0.01) and TLR-4 (*p* ≤ 0.001) blockade (Figure 4A). IL-6 levels were also markedly elevated by the lysate (*p* ≤ 0.001) and LPS (*p* ≤ 0.001), but not by Pam3CSK4. Neutralization of TLR-2 and TLR-4 significantly reduced IL-6 levels by *P. gingivalis* lysate (*p* ≤ 0.05) (Figure 4B). Similarly, IL-8 secretion increased significantly in response to *P. gingivalis* (*p* ≤ 0.0001) and LPS (*p* ≤ 0.0001), while Pam3CSK4 had no effect. TLR-2 and TLR-4 blockade significantly reduced IL-8 production by *P. gingivalis* lysate (Figure 4C). TNF-α secretion was significantly increased by *P. gingivalis* lysate (*p* ≤ 0.001), Pam3CSK4 (*p* ≤ 0.05), and LPS (*p* ≤ 0.0001) compared to unstimulated controls. Notably, TLR-2 blockade did not reduce TNF-α secretion; instead, it increased it further (*p* ≤ 0.05) compared to the lysate-only condition, suggesting a possible compensatory mechanism. TLR-4 inhibition had no significant effect (Figure 4D). IL-10, in contrast, showed a modest but significant increase in response to LPS (*p ≤* 0.01) compared to unstimulated controls, while *P. gingivalis* lysate and Pam3CSK4 had no effect. Neither TLR-2 nor TLR-4 blockade altered IL-10 levels significantly (Figure 4E). Control experiments using the IgA2 isotype antibody confirmed that cytokine responses were specifically dependent on TLR signaling, as no significant changes were observed in this condition.

These data demonstrate that *P. gingivalis* lysate induces a potent proinflammatory cytokine profile in human placental explants, mediated primarily through TLR-2 and TLR-4, with distinct roles depending on the cytokine involved.

## 3. Discussion

This study demonstrates that *P. gingivalis* lysate elicits a robust proinflammatory response in HPEs, mediated primarily through TLR-2 and TLR-4. This activation leads to canonical NF-κB signaling, release of inflammatory cytokines, and tissue-level changes characteristic of placental damage. Importantly, our work represents, to our knowledge, the first experimental analysis directly assessing the response of the human placental barrier to *P. gingivalis* components using villous explants that preserve histological architecture and trophoblast–stroma interactions. While previous studies focused mainly on detecting bacterial DNA or antigens in placental tissue [12,22] or relied on animal models [16], our findings provide novel mechanistic insight into innate immune activation at the placental barrier and establish a potential link between maternal periodontitis and adverse pregnancy outcomes.

We used *P. gingivalis* ATCC 33277®, a well-characterized laboratory strain that expresses major virulence factors, including LPS, gingipains, fimbriae, and peptidylarginine deiminase (PPAD) [7,9,10,23,24]. This strain is widely employed in immunological and pathogenicity studies due to its genetic stability and ability to elicit consistent host responses, minimizing variability inherent to clinical isolates [10]. Exposure to bacterial lysates allowed us to mimic the effects of microbial products without the confounding influence of live infection, thereby isolating the contribution of bacterial components to placental immune activation, making them suitable for in vitro immunomodulatory studies [25,26].

Our findings show that *P. gingivalis* lysate induces significant tissue injury in HPEs, including trophoblast detachment and stromal disorganization, even at low concentrations (10 µg/mL). Collagen degradation was evident at higher concentrations (≥50 µg/mL), as demonstrated by reduced Masson’s trichrome and Picrosirius Red staining. These findings are consistent with the known proteolytic effects of gingipains on extracellular matrix components [27,28], which may compromise the placental barrier. Moreover, gingipains indirectly contribute to tissue damage by activating latent host matrix metalloproteinases (MMPs) and the inactivation of host proteinase inhibitors [28]. *P. gingivalis* itself has been shown to induce MMPs in human gingival fibroblasts [29]. The histopathological changes are consistent with the alterations induced by other relevant pathogens that can be transmitted congenitally, such as *Toxoplasma gondii* [17,30].

Although *P. gingivalis* primarily signals through TLR-2, our results reveal that stimulation with *P. gingivalis* lysate significantly reduced membrane-bound TLR-2 protein levels in HPEs, while concurrently increasing soluble TLR-2 (sTLR-2) concentrations in the culture supernatants. This inverse relationship suggests that TLR-2 may undergo proteolytic cleavage or alternative splicing, releasing its soluble form as a regulatory mechanism to inflammatory stimuli [21]. sTLR-2 has been shown to function as a decoy receptor, binding pathogen-associated molecular patterns (PAMPs) and dampening TLR-2-mediated proinflammatory signaling [31]. The observed modulation may result from receptor shedding, likely mediated by bacterial gingipains or host-derived metalloproteinases, a mechanism previously described in other infectious contexts [21]. In contrast, TLR-4 protein expression remained unchanged, underscoring a preferential targeting of TLR-2 in placental tissue. This modulation of surface PRRs may shift the threshold for innate immune activation, potentially influencing the delicate balance between immune tolerance and inflammation during pregnancy.

Activation of Toll-like receptors by microbial components typically triggers intracellular signaling cascades, most notably the canonical NF-κB pathway, which governs the transcription of numerous proinflammatory genes and is a central mediator of innate immune responses [19,32]. Our study observed that *P. gingivalis* lysate and the TLR-2 agonist Pam3CSK4 triggered phosphorylation of IκBα, indicating activation of this pathway. These results were confirmed by immunoblotting and immunohistochemistry, which showed increased phosphorylation of IκBα predominantly in trophoblast cells. Functional assays demonstrated that TLR-2 blockade significantly reduced *P. gingivalis*-induced NF-κB activation, whereas TLR-4 inhibition did not affect this response, despite effectively suppressing NF-κB activation induced by canonical LPS stimulation. These findings identify TLR-2 as the primary sensor of *P. gingivalis* in placental tissues, consistent with previous studies demonstrating that TLR-2 is dominant in recognizing Gram-negative oral pathogens at mucosal and epithelial barriers [18]. Moreover, our observations align with previous reports demonstrating that *P. gingivalis* LPS induces NF-κB activation via IκBα degradation in human aortic endothelial cells [33] and enhances p-IκBα expression in co-cultures of human gingival fibroblasts and THP-1 monocytes [29]. Notably, NF-κB activation within the placenta has been associated with pathological inflammation, including preterm labor and chorioamnionitis, conditions in which increased numbers of NF-κB–positive immune cells have been identified in placental tissues [30,34].

The strong upregulation of IL-1β, IL-6, IL-8, and TNF-α in HPE supernatants following *P. gingivalis* lysate exposure further confirmed the inflammatory response. These cytokines play key roles in leukocyte recruitment, vascular permeability, and local inflammation, while their dysregulation has been implicated in preterm labor and other obstetric complications [15,20]. Neutralizing TLR-2 and TLR-4 effectively reduced IL-1β, IL-6, and IL-8 secretion, confirming their receptor-mediated origin. However, TNF-α secretion was paradoxically increased upon TLR-2 blockade, suggesting a compensatory or regulatory mechanism, possibly involving alternative PRRs or feedback loops. Interestingly, gingipains have been demonstrated to affect cytokine signaling networks and modulate the production of proinflammatory mediators, including the here studied IL-1β, IL-6, IL-8, and TNF-α [28], explaining the observed tissue damage. IL-10, on the other hand, was modestly increased but not modulated by TLR blockade, suggesting it may represent a parallel regulatory mechanism insufficient to counterbalance the strong proinflammatory signals.

Nevertheless, some limitations should be considered when interpreting our results. First, the explants were obtained from term placentas, which may not fully reflect the immunological conditions of the first trimester, a critical period for infection susceptibility, implantation, and early pregnancy maintenance. Second, while HPEs explants preserve tissue architecture and local cell–cell interactions, they do not capture the systemic contributions of maternal and fetal immune responses, such as leukocyte trafficking or endocrine regulation, which are known to modulate placental immunity in vivo [17]. Future studies should integrate models using earlier gestational tissues together with in vivo or organ-on-chip approaches that incorporate maternal and fetal compartments to better recapitulate the complexity of host–pathogen interactions at the maternal–fetal interface.

## 4. Materials and Methods

### 4.1. Bacterial Strains and Lysate Preparation

*P. gingivalis* (ATCC 33277^®^, Manassas, VA, USA) [35] was cultured anaerobically on blood agar with hemin/menadione and in BHI broth (Oxoid, Basingstoke, UK). Bacteria were harvested, washed, and lysed by freeze-thaw cycles and sonication. Lysate sterility was confirmed, and protein was quantified by BCA assay (Thermo Fisher Scientifi Inc., Waltham, MA, USA). LPS content was analyzed by TRIzol (Thermo Fisher Scientifi Inc.^®^, Waltham, MA, USA) extraction and SDS-PAGE with silver staining. Coomassie-stained SDS-PAGE (Thermo Fisher Scientifi Inc.^®^, Waltham, MA, USA), verified protein integrity, and gingipain activity were assessed via DL-BAPNA assay (Sigma Aldrich^®^, Burlington, MA, USA) [36] (Supplementary File S1).

### 4.2. Human Placental Explants (HPEs) Collection and Culture

Term placentas (≥38 weeks) from healthy donors undergoing elective cesarean at Hospital San José were collected with informed consent and ethical approval (Universidad de Chile, No. 042-2022). Cases with fetal or maternal pathology were excluded. Villous tissue was obtained from the central part of central cotyledons, processed within 30 min post-delivery, dissected into 0.5 cm^3^ fragments, and cultured in RPMI 1640 (Gibco, New York, NY, USA) with 5% FBS and 1% penicillin-streptomycin at 37 °C in 5% CO_2_ [17].

### 4.3. Experimental Design and Treatments

HPEs were exposed to *P. gingivalis* lysates at concentrations ranging from 10 to 200 µg/mL for 24 h to determine the optimal stimulation condition. We selected 50 µg/mL lysate concentration for subsequent experiments since this is the minimal concentration that induces histopathological damage in the trophoblast and villous stroma. Control explants were incubated with equivalent volumes of PBS. To investigate the protein expression of Toll-like receptors (TLRs), HPEs were pretreated with neutralizing antibodies against TLR-2 and TLR-4 (5 µg/mL, Invivogen^®^, San Diego, CA, USA) for 30 min before lysate stimulation. As positive controls, Pam3CSK4 (1 µg/mL; (Sigma Aldrich^®^, Burlington, MA, USA)) and *E. coli* LPS (100 ng/mL (Sigma Aldrich^®^, Burlington, MA, USA)) were employed to activate TLR-2 and TLR-4 signaling pathways, respectively. For NF-κB activation, HPEs were incubated for 10 min in the presence and absence of 50 µg/mL lysate. This signaling pathway is activated after 10 min stimulus in HPEs, as shown by us previously [19].

### 4.4. Histological and Immunohistochemical Analysis

HPEs were fixed in 4% paraformaldehyde, paraffin-embedded, sectioned (3–5 µm), and stained with H&E for histology or with Masson’s trichrome and Picrosirius Red for collagen (dyes were acquired from Merck^®^, Darmstadt, Germany). Immunohistochemistry was performed using antibodies against TLR-2, TLR-4, and NF-κB p65 (Cell Signaling^®^, Danvers, MA, USA) after antigen retrieval. Detection was via HRP-conjugated secondary antibodies and DAB (Cell Signaling^®^, Danvers, MA, USA). Staining intensity and immunopositivity were quantified using ImageJ 1.54p, and histological damage was scored semi-quantitatively across ten random fields according to the scores depicted in Table 1 [37].

### 4.5. Western Blot Analysis

HPEs were homogenized in lysis buffer with protease inhibitors, sonicated, and centrifuged. Supernatants were quantified via BCA assay. Proteins (50 µg) were denatured, separated by SDS-PAGE, and transferred to PVDF (Thermo Fisher Scientifi Inc.^®^, Waltham, MA, USA) membranes. Western blotting was performed for TLR-2, TLR-4, phospho-IκBα, total IκBα, and GAPDH using specific antibodies (Cell Signaling^®^, Danvers, MA, USA). Detection used HRP-conjugated secondary antibodies and chemiluminescence (Cell Signaling^®^, Danvers, MA, USA). Band intensities were analyzed with ImageJ 1.54p and expressed relative to GAPDH [19,38].

### 4.6. Cytokine Quantification by ELISA and Cytometric Bead Array

HPE culture supernatants were cleared by centrifugation (500× *g*, 20 min, 4 °C). IL-1β, IL-6, IL-8, IL-10, and TNF-α levels were measured using BD Cytometric Bead Array (BD Bioscience, Franklin Lakes, NJ, USA) and analyzed with FACSuite™ (BD Bioscience, Franklin Lakes, NJ, USA) and FCAP Array (BD Bioscience, Franklin Lakes, NJ, USA). Soluble TLR-2 was quantified by ELISA (Invitrogen^®^; detection limit 0.32 ng/mL). Cytokine levels were normalized to the total protein content in the supernatant. No significant differences in protein concentration were observed across experimental conditions.

### 4.7. Statistical Analysis

Data are presented as mean ± standard deviation (SD). Statistical comparisons between groups were performed using one-way ANOVA followed by Tukey’s post hoc test. Differences were considered statistically significant at *p* < 0.05. GraphPad Prism 9.0 software was used for all analyses.

## 5. Conclusions

**Figure 5:**
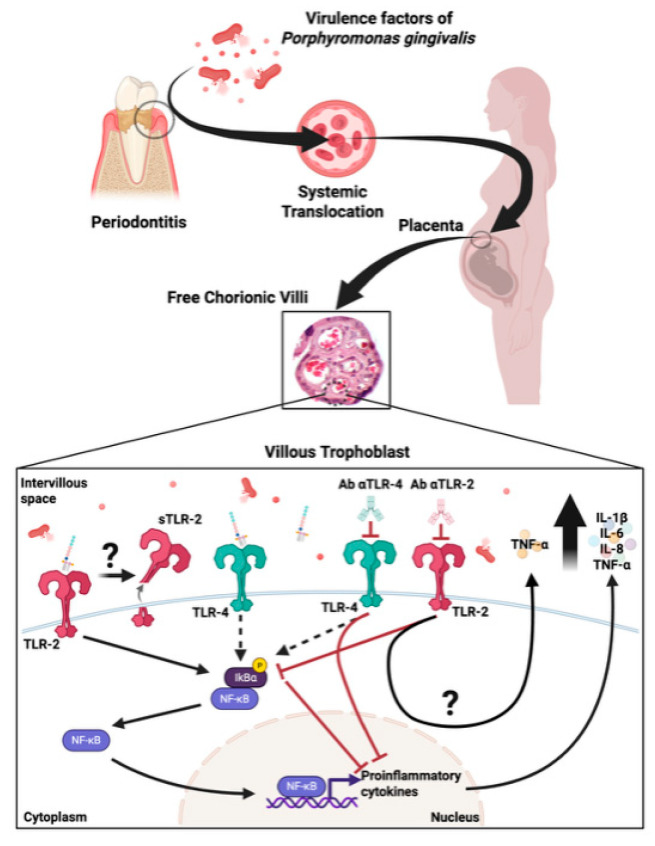
Proposed model of TLR-mediated placental immune response to *P. gingivalis* lysate: Schematic summary of the proposed mechanism by which *P. gingivalis* lysate activates innate immune responses in HPEs. Upon stimulation, bacterial components interact with trophoblast-expressed TLR-2 and, to a lesser extent, TLR-4, triggering canonical NF-κB signaling and proinflammatory cytokine production (IL-1β, IL-6, IL-8, TNF-α). Concurrently, TLR-2 surface expression is reduced, and soluble TLR-2 (sTLR-2) is released into the extracellular space, potentially acting as a decoy receptor to modulate the inflammatory response. This local activation may contribute to placental barrier dysfunction and adverse pregnancy outcomes such as preterm birth or fetal growth restriction. The figure encapsulates the placental innate immune dynamics in response to oral pathogen-derived products at the maternal-fetal interface.

Our findings show that *P. gingivalis* lysate creates a proinflammatory placental environment through TLR-2/4–mediated NF-κB activation and cytokine release, potentially compromising placental integrity and contributing to adverse pregnancy outcomes. Our results highlight the placental barrier as an active immune site (Figure 5). Future studies should confirm these effects in vivo and explore how periodontal treatment may mitigate related obstetric risks.

## Figures and Tables

**Table 1:** Scores for tissue damage analysis.

Score	Histopathology Damage
1	Attached trophoblast, intact fetal connective tissue
2	Slight trophoblast detachment and/or fetal connective tissue disorganization
3	Almost complete trophoblast detachment and/or fetal connective tissue disorganization
4	Complete trophoblast detachment and disorganization or destruction of the fetal connective tissue

## Data Availability

All data supporting the findings are available within the paper and its Supplementary Information.

## Supplementary Materials

The following supporting information can be downloaded at: https://www.mdpi.com/article/10.3390/ijms26199558/s1.

## Abbreviations

The following abbreviations are used in this manuscript

HPEs	Human placental explants
*P. gingivalis*	*Porphyromonas gingivalis*
NF-kB	Nuclear Factor kappa B
TLRs	Toll-like Receptors
TLR-2	Toll-like Receptor-2
TLR-4	Toll-like Receptor-4
sTLR2	Soluble TLR-2
IL1β	Interleukin-1 beta
IL-6	Interleukin-6
IL-8	Interleukin-8
IL-10	Interleukin-10
TNF-α	Tumor necrosis Factor alpha
DNA	Deoxyribonucleic acid
LPS	Lipopolysaccharides
IVS	Intervillous space
STB	Syncytiotrophoblast
CTB	Cytotrophoblast
VS	Villous stroma
PRRs	Pattern recognition receptors
PAMPs	Pathogen-associated molecular patterns
SD	Standard deviation
Pam3CSK4	N-Palmitoyl-S-[2,3-bis(palmitoyloxy)-(2RS)-propyl]- [R]-cysteinyl-[S]- seryl-[S]-lysyl-[S]-lysyl-[S]-lysyl-[S]-lysine
*E. coli*	*Escherichia coli*
p-IκBα	Phospho-I-kappa-B-alpha
IκBα	I-kappa-B-alpha
IgA2	Immunoglobulin A2
PPAD	Peptidylarginine deiminase
ATCC	American Type Culture Collection
BHI	Brain Heart Infusion
SDS-PAGE	Sodium dodecyl sulfate polyacrylamide gel electrophoresis
DL-BAPNA	Nα-Benzoyl-DL-arginine 4-nitroanilide hydrochloride
FBS	Fetal bovine serum
CO_2_	Carbon dioxide
H&E	Hematoxylin and eosin
HRP	Horseradish peroxidase
DAB	Diaminobenzidine
BCA	Bicinchoninic acid
PVDF	Polyvinylidene fluoride
GAPDH	Glyceraldehyde-3-phosphate Dehydrogenase
