# Supplementary material for: Porphyromonas gingivalis Lysate Induces TLR-2/4-Dependent NF-κB Activation and Inflammatory Damage in the Human Placental Barrier
Source: Int J Mol Sci. 2025 Sep 30;26(19):9558. doi: 10.3390/ijms26199558 (PMC12524479; doi:10.3390/ijms26199558)
Supplement: Supplementary file 1 [file ijms-26-09558-s001.zip › ijms-3897813-supplementary.pdf]

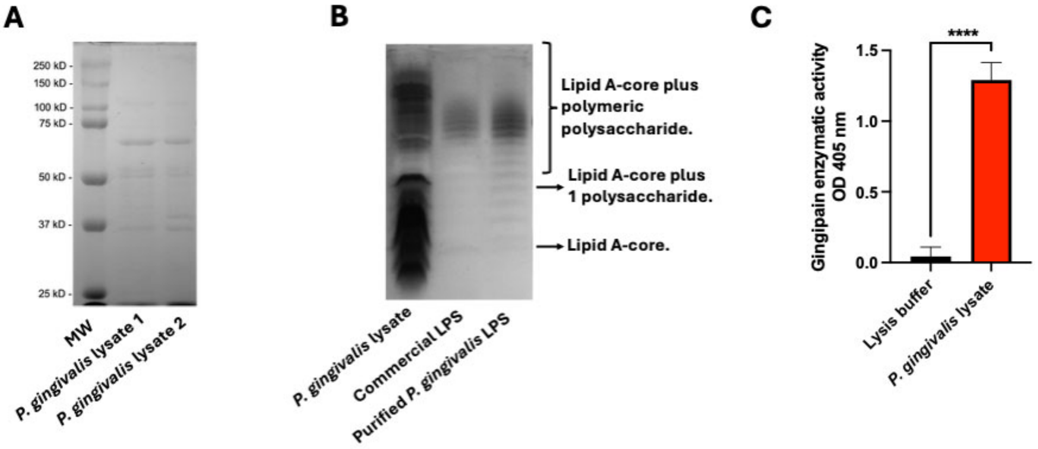

Supplementary Figure S1. Characterization of *P. gingivalis* Lysate reveals presence of LPS and Gingipain enzymatic Activity: To assess the protein integrity, presence of LPS, and enzymatic content of the *P. gingivalis* lysate used in this study, 45 μg of total protein from two independent lysate preparations were separated by SDS-PAGE and visualized with Coomassie Brilliant Blue staining (A). The gel revealed a diverse protein profile with multiple bands of varying molecular weights, confirming the presence of intact and heterogeneous bacterial proteins. The lysate was characterized for lipopolysaccharide (LPS) content via a modified TRIzol® extraction protocol, visualized by SDS-PAGE with silver staining, and compared to commercial LPS (Invivogen®). The lysate exhibited LPS bands corresponding to the lipid A-core structure with varying degrees of polymeric polysaccharide, confirming preservation of LPS components after processing (B). To evaluate gingipain activity in the lysate, a chromogenic assay was performed using a synthetic substrate specific for arginine-specific protease activity. The lysate (C) showed increased optical density at 405 nm across lysate concentrations, confirming that gingipain enzymes remain functionally active in the preparation. Data represent the mean ± SD of three independent experiments. ****$p < 0.0001$ versus control.
